# Impact of Lipodystrophy on the prevalence and components of metabolic syndrome in HIV-infected patients

**DOI:** 10.1186/1471-2334-11-246

**Published:** 2011-09-20

**Authors:** Paula Freitas, Davide Carvalho, Selma Souto, Ana Cristina Santos, Sandra Xerinda, Rui Marques, Esteban Martinez, António Sarmento, José Luís Medina

**Affiliations:** 1 Endocrinology, Diabetes and Metabolism Department of Centro Hospitalar São João, E.P.E., University of Porto Medical School, Alameda Hernani Monteiro, 4200 - 319 Porto, Portugal; 2 Clinical Epidemiology, Predictive Medicine and Public Health Department, University of Porto Medical School, Porto, Portugal and University of Porto Institute of Public Health, Alameda Hernani Monteiro, 4200 - 319 Porto, Portugal; 3 Infectious Disease Department of Centro Hospitalar São João, E.P.E., Alameda Hernani Monteiro, 4200 - 319 Porto, Portugal; 4 Infectious Diseases Department, Hospital Clinic, University of Barcelona, C/Villarroel 170, Barcelona 08036, Spain; 5 Infectious Disease Department of Centro Hospitalar São João, E.P.E, University of Porto Medical School, Alameda Hernani Monteiro, 4200 - 319 Porto, Portugal

**Keywords:** Metabolic syndrome, Cardiovascular Risk, Lipodystrophy, HIV infection

## Abstract

**Background:**

In HIV-infected patients, combination antiretroviral therapy (cART) is associated with clinical lipodystrophy (CL) and metabolic abnormalities (MA). This study aimed to evaluate the prevalence of the metabolic syndrome (MS) and its components, and to determine whether patients with or without CL had a different prevalence of MA.

**Methods:**

We evaluated 345 HIV-infected patients on cART using two different MS definitions (NCEP-ATPIII-2005 and IDF-2005) and the Framingham risk score.

**Results:**

CL was present in 58.7% of the patients. The prevalence of the MS was 52.2% (ATPIII) and 43.2% (IDF), and it was not significantly different between patients with (W) or without (WT) CL, regardless of the definition used (ATPIII WCL 52.9% vs WT CL 51.1%; p = 0.738; IDF WCL 41.3% vs WTCL 46.0%; p = 0.379). Moderate concordance was observed between the 2 definitions (kappa = 0.484; p < 0.001) and after gender stratification there was good concordance in women (kappa = 0.759; p < 0.001). Patients with CL had lower waist circumference and HDL-C and higher triglycerides levels. In women, CL was significantly associated with MS, hypertriglyceridemia and low HDL cholesterol independently of age, cART and BMI. Patients with CL had a significantly higher risk of coronary heart disease at 10 years, measured by the Framingham risk score, than patients without CL. Those with CL and with MS had higher frequencies of moderate and high risk categories than those without MS.

**Conclusions:**

The prevalence of the MS was high in these HIV-infected patients with an age average of 40 years and this finding could explain why HIV patients have an increased risk for cardiovascular disease (CVD).

## Background

Combination antiretroviral therapy (cART) has changed the course of HIV infection, leading to a significant reduction in AIDS related morbidity and mortality [1]. However, cART is known to be associated with changes in fat distribution (lipodystrophy) and metabolic abnormalities, including insulin resistance, dyslipidemia, and increased blood pressure (BP) [2,3].

The Metabolic syndrome (MS), a common condition in the general population, refers to a constellation of cardiovascular disease (CVD) risk factors, including increased waist circumference (WC), glucose and lipid metabolism disorders, and hypertension [4,5]. Each of these risk factors individually increases cardiovascular risk, but the MS itself has shown to be a powerful independent risk factor for cardiovascular morbi-mortality and diabetes [6]. It is still controversial if the MS improves the identification of high risk individuals [7-9]. HIV-infected lipodystrophic patients share several components of the MS. The prevalence of the MS in HIV-infected patients has been reported to range from 4.4 to 45.4% [9-23].

HIV-lipodystrophy is considered to be an adverse effect of cART, not limited to a specific drug or class of drugs [24]. It seems to affects up to half or even more HIV-infected patients receiving cART [25]. However, few studies address the prevalence of the MS in patients with lipodystrophy [12,14,17].

The aim of this study was to evaluate the prevalence of the MS and of its five individual components in HIV-infected patients on cART and to determine whether patients with or without CL had a different prevalence of MA.

## Methods

### Subjects

As part of a cross-sectional study, 345 non-institutionalized, HIV-infected Caucasian adults (239 males), on cART referred from the Infectious Diseases to the Endocrinology Out-patient Clinic of Hospital São João due to lipodystrophy or any metabolic disorders were included. Only patients on cART were included, since lipodystrophy is mainly related to ART drugs. The study protocol was approved by the Hospital's Ethical Committee and all patients provided informed consent.

### Clinical assessment

For each patient the following information was collected, using a standardized protocol: demographic data (age, gender), duration of HIV infection, history of diabetes, hypertension, use of antidiabetic, antihypertensive or lipid-lowering drugs and duration of cART. Weight, height, WC and resting BP were measured as previously described [26]. Body weight was measured using TANITA (Tanita^®^, model TBF 300) scale and height was measured to the nearest centimetre in the standing position using a wall stadiometer (Holtain Limited Crymych, Dyfed^®^).

Clinical lipodystrophy (CL) and central fat accumulation or abdominal prominence, were defined as previously described [27]. All clinical assessments were performed by the same practitioner (PF). Patients were classified in 4 different groups according to the presence or absence of either lipoatrophy or abdominal prominence: group 1- **No lipodystrophy **(patients without clinical lipoatrophy and without abdominal prominence); group 2- **Isolated central fat accumulation **(patients without clinical lipoatrophy and with abdominal prominence); group 3- **Isolated lipoatrophy **(patients with clinical lipoatrophy and without abdominal prominence); group 4- **Mixed forms of lipodystrophy **(patients with clinical lipoatrophy and with abdominal prominence).

A venous blood sample was drawn after a 12-hour overnight fast. All the samples were analyzed at the central laboratory of our hospital. Plasma glucose, total cholesterol, HDL cholesterol (HDL-C) and triglycerides were determined using automatic standard routine enzymatic methods. Hepatitis C was diagnosed by HCV-Ab serostatus. The CD4+ cell count was determined by flow cytometry and plasma RNA-VIH was measured by a quantitative reverse transcriptase polymerase chain reaction, which had a lower limit of detection of 50 copies/mL.

### Metabolic Syndrome definitions

Two different MS definitions were used: the Third Report of the National Cholesterol Education Program (NCEP) Expert Panel on Detection, Evaluation, and Treatment of High Blood Cholesterol in Adults (ATPIII) modified by American Heart Association/National Heart, Lung, and Blood Institute Scientific Statement (AHA/NHLBI) in 2005 [28] and the International Diabetes Federation (IDF) definition [29].

The Framingham cardiovascular risk score was calculated for all patients using the Framingham methodology as reported by Wilson. It incorporates age, gender, systolic blood pressure, lipids, glucose, and smoking status into a model that predicts the probability of coronary heart disease risk at 10 years [30].

### Statistical analysis

Data were presented as mean and standard deviation (SD) for quantitative variables when normally distributed. When the variable distribution was different than the normal, it was expressed as median and respective 25^th ^and 75^th ^percentiles. For comparison between quantitative variables Student-t test and the Mann-Whitney test were used when appropriate. Categorical variables were described as counts and proportions, and compared using the chi-square or Fisher's exact test. Kappa coefficients were estimated to analyze the statistical agreement between ATP III as modified by the AHA/NHLBI and IDF definitions of MS.

Odds ratios and their respective 95% confidence intervals (CI) were computed using unconditional logistical regression models. The association between lipodystrophy and the presence of MS and its individual components was estimated after adjustment for sex, age, cART duration and BMI, whenever appropriate.

Statistical analysis was performed using the SPSS version 17.0 software (SPSS Inc., Chicago, Illinois, USA). All probabilities were two tailed and p values < 0.05 were regarded as significant.

## Results

Of the 345 HIV-1 infected patients on cART included in this study, 58.7% presented CL. Table 1 shows the characteristics of the study sample according to the presence of CL. Patients with CL were more frequently male, older, had longer duration of infection, longer duration of cART and lower weight, BMI and WC. Also, this group of patients had significantly higher CD4+ cell counts and a significantly higher prevalence of virological suppression. No significant differences in HIV risk factors or in cART regimen composition were found between patients with or without CL. The prevalence of hepatitis C co-infection was 28.8%, without significant differences between patients with or without CL. No significant differences were found in smoking history (never, current or former) between patients with or without CL, however, patients who were current smokers had the highest frequency of low and normal weight and those who never smoked had the highest frequency of overweight and obesity (data not shown). No significant difference was found in fasting glucose according to smoking history (data not shown). According to the presence of CL, no differences were found in systolic and diastolic BP, glucose and total cholesterol levels. However, patients with CL had lower HDL-C and higher triglycerides plasma levels (Table 1).

**Table 1:** Sample characteristic according to the presence of clinical lipodystrophy

	Without CL	With CL	P
n (%)	139 (39.6)	206 (58.7)	

Sex [n(%)]			
Male	85 (61.2)	154 (74.8)	0.007
Female	54 (38.8)	52 (25.2)	

Age [years, median (25^th ^and 75^th ^percentiles)]	42.0 (34.0-51.0)	45.0 (39.0-54.0)	0.006

Duration of HIV infection [years, median (25^th ^and 75^th ^percentiles)]	6.0 (4.0-9.0)	9.0 (6.0-11.0)	< 0.001

cART [years, median (25th and 75th percentiles)]	4.0 (2.0-7.0)	8.0 (5.0-10.0)	< 0.001

Weight [Kg, mean (sd)]	73.6 (14.1)	66.2 (12.4)	< 0.001

Height [m, mean (sd)]	1.65 (0.09)	1.66 (0.09)	0.430

BMI [(kg/m^2^), mean (sd)]	27.0 (5.0)	24.0 (3.9)	< 0.001

Waist circumference [cm, mean (sd)]	95.4 (13.0)	89.5 (10.5)	< 0.001

Systolic BP [mmHg, median (25^th ^and 75^th ^percentiles)]	120.0 (110.0-135.0)	120.0 (110.0-135.0)	0.871

Diastolic BP [mmHg, median (25^th ^and 75^th ^percentiles)]	80.0 (70.0-80.0)	80.0 (70.0-80.0)	0.765

CD4 cell count [cells/mm3, median (25^th ^and 75^th ^percentiles)]	446.5 (291.5-633.8)	544.0 (362.0-749.0)	0.008

HIV RNA (< 50) [n (%)]	94 (82.5)	163 (91.1)	0.029

Hepatitis C co-infection [n (%)]	31 (25)	58 (31.4)	0.227

HIV risk factor [n (%)]			

Intravenous drug user	28 (24.6)	52 (30.6)	

Homosexual contact	10 (8.8)	19 (11.2)	

Heterosexual contact	75 (65.8)	90 (52.9)	

Others	1 (0.9)	9 (5.3)	0.073

CDC [n (%)]			

A	62 (54.9)	94 (53.4)	

B	4 (3.5)	0 (0.0)	

C	47 (41.6)	82 (46.6)	0.037

ART [n (%)]			

IP	67 (61.5)	98 (55.4)	0.310


NNRTI	47 (43.1)	83 (46.9)	0.534

NRTI	104 (95.4)	175 (98.9)	0.066

Smoking history [n (%)]			

Never	60 (43.5)	70 (34.8)	

Current	56 (40.6)	93 (46.3)	

Former	22 (15.9)	38 (18.9)	0.272

Glucose [mg/dL, median (25^th ^and 75^th ^percentiles)]	91.0 (84.0-108.0)	94.5 (863.0-117.0)	0.074

Total cholesterol [mg/dL, mean (sd)]	229.5 (59.8)	220.7 (59.5)	0.182

HDL- cholesterol [mg/dL, median (25^th ^and 75^th ^percentiles)]	48.0 (37.0-55.0)	43.0 (35.0-53.0)	0.047

Triglycerides [mg/dL, median (25^th ^and 75^th ^percentiles)]	192.0 (126.0-323.0)	231.0 (149.8-365.8)	0.016

Metabolic syndrome ATP III [n (%)]	71 (51.1)	109 (52.9)	0.738

Metabolic syndrome IDF [n (%)]	64 (46.0)	85 (41.3)	0.379

Metabolic syndrome features - ATP III			
High blood pressure	61 (43.9)	87 (42.4)	0.790
Hypertriglyceridemia	98 (70.5)	177 (85.9)	< 0.001
Low HDL cholesterol	93 (66.9)	160 (77.7)	0.027
High waist circumference	58 (41.7)	46 (22.3)	< 0.001
High fasting glucose	51 (36.7)	84 (40.8)	0.446

Number of metabolic syndrome features ATP III			
0	6 (4.3)	9 (4.4)	0.242
1	25 (18.0)	19 (9.3)	
2	37 (26.6)	69 (33.7)	
3	31 (22.3)	52 (25.4)	
4	31 (22.3)	42 (20.5)	
5	9 (6.5)	14 (6.8)	

Metabolic syndrome features - IDF			
High blood pressure	61 (43.9)	87 (42.4)	0.790
Hypertriglyceridemia	98 (70.5)	177 (85.9)	< 0.001
Low HDL cholesterol	93 (66.9)	160 (77.7)	0.027
High waist circumference	87 (62.6)	94 (45.6)	0.002
High fasting glucose	51 (36.7)	80 (38.8)	0.687

Number of metabolic syndrome features IDF			
0	5 (3.6)	9 (4.4)	0.263
1	19 (13.7)	17 (8.3)	
2	35 (25.2)	51 (24.9)	
3	32 (23.0)	63 (30.7)	
4	35 (25.2)	39 (19.0)	
5	13 (9.4)	26 (12.7)	

Note - CL = Clinical lipodystrophy, MS = Metabolic syndrome; ATPIII = Third Report of the National Cholesterol Education Program Expert Panel on Detection, Evaluation, and Treatment of High Blood Cholesterol in Adults; IDF = International Diabetes Federation; BMI = body mass index.

Regarding BMI distribution, men and women with CL, were more frequently classified as normal when compared to those without CL. Those without CL were more frequentely classified as overweight (Table 2).

**Table 2:** BMI by gender and clinical lipodystrophy

	Men		Women	
**BMI** **[kg/m^2^, (n (%)]**	**Without CL**	**With CL**	**Without CL**	**With CL**

< 18.5	1 (1.2)	9 (5.8)	0 (0.0)	8 (15.4)
≥18.5 and < 25	38 (44.7)	88 (57.1)	16 (30.2)	25 (48.1)
≥25 and < 30	31 (36.5)	47 (30.5)	20 (37.7)	15 (28.8)
≥30	15 (17.6)	10 (6.5)	17 (32.1)	4 (7.7)
p value	0.009		< 0.001	

Note - CL = Clinical lipodystrophy; BMI = body mass index.

### Metabolic syndrome

In our sample, the prevalence of MS was 52.2% and 43.2%, according to ATPIII and IDF criteria, respectively. No significant differences in the prevalence of the MS were observed in patients with or without CL, regardless of the definition used (table 3). Moderate concordance was observed between the 2 definitions (kappa = 0.484; p < 0.001). After gender stratification, moderate agreement was observed in men (kappa = 0.374; p < 0.001), and good agreement in women (kappa = 0.759; p < 0.001). Using both MS criteria, no significant differences in the prevalence of CL were found between patients with and without MS [[with MS 60.6% (109 patients) vs without MS 58.8% (97 patients); p = 0.822 for ATPIII] and [with MS 57.0% (85 patients) vs without MS 61.7% (121 patients); p = 0.442 for IDF]]. No significant differences according to gender and presence of CL were observed regarding the prevalence of the MS (Table 3). However, men and women with lipodystrophy had a significantly different prevalence of the MS when stratified by BMI classes, regardless the criteria used to define the MS (Table 4). Men and women with CL presented a significantly higher prevalence of the MS in the normal and underweight class (Table 4). A significantly higher prevalence of hypertriglyceridemia, low HDL-C levels and lower prevalence of abdominal obesity were observed in patients with CL. No differences were found in the number of the MS features according to ATPIII and IDF criteria in patients with and without CL (Table 1).

**Table 3:** Metabolic syndrome prevalence according to ATP III and IDF criteria in the total of patients and by gender and presence of clinical lipodystrophy

MS Criteria	Total of patients	Without CL	With CL	P
ATP III [n, (%)]				
Total	180 (52.2%)	71 (51.1%)	109 (52.9%)	0.738
Men	130 (54.4%)	46 (54.1%)	84 (54.5%)	0.949
Women	50 (47.2%)	25 (46.3%)	25 (48.1%)	0.854

IDF [n, (%)]				
Total	149 (43.2%)	64 (46.0%)	85 (41.3%)	0.379
Men	88 (36.8%)	36 (42.4%)	52 (33.8%)	0.188
Women	61 (57.5%)	28 (51.9%)	33 (63.5%)	0.227

Note - CL = Clinical lipodystrophy MS = Metabolic syndrome; ATPIII = Third Report of the National Cholesterol Education Program Expert Panel on Detection, Evaluation, and Treatment of High Blood Cholesterol in Adults; IDF = International Diabetes Federation; BMI = body mass index.

**Table 4:** MS prevalence by BMI, gender and clinical lipodystrophy according to ATPIII criteria and IDF

	Men		Women	
	**Without CL**	**With CL**	**Without CL**	**With CL**

ATPIII criteria				

Classes of BMI [kg/m^2^; (n (%)]				

< 25	14 (30.4%)	41 (48.8%)	3 (12.5%)	11 (44.0%)
≥25 < 30	19 (41.3%)	33 (39.3%)	9 (37.5%)	10 (40.0%)
≥30	13 (28.3%)	10 (11.9%)	12 (50.0%)	4 (16.0%)
p value	0.002	< 0.001	0.012	0.008

IDF criteria				

< 25	4 (11.1%)	11 (21.2%)	5 (18.5%)	16 (48.5%)
≥25 < 30	18 (50.0%)	32 (61.5%)	10 (37.0%)	13 (39.4%)
≥30	14 (38.9%)	9 (17.3%)	12 (44.4%)	4 (12.1%)
p value	< 0.001	< 0.001	0.084	0.012

Note - CL = Clinical lipodystrophy; MS = Metabolic syndrome; ATPIII = Third Report of the National Cholesterol Education Program Expert Panel on Detection, Evaluation, and Treatment of High Blood Cholesterol in Adults; IDF = International Diabetes Federation; BMI = body mass in

Regarding the aggregate of the MS criteria using the ATP definition, the most frequent aggregate number was 2, except for men without CL. On the other hand, using the IDF definition, the most frequent aggregate number was 3, except for women without CL (table 5 and 6). No significant increase of MS prevalence with age was observed in patients with or without CL (data not shown).

**Table 5:** Frequency of each criterion of MS according to ATP III and IDF in men with and without clinical lipodystrophy

Men								
**ATPIII**					**IDF**			

**MS criteria**	**Total**	**Without CL**	**With CL**	**p value**	**Total**	**Without CL**	**With CL**	**p value**

WC [n, (%)]	43 (18.0%)	21 (24.7%)	22 (14.3%)	0.045	98 (41.0%)	42 (49.4%)	56 (36.4%)	0.050
TG [n, (%)]	197 (82.4%)	65 (76.5%)	132 (85.7%)	0.072	197 (82.4%)	65 (76.5%)	132 (85.7%)	0.072
HDL [n, (%)]	181 (75.7%)	62 (72.9%)	119 (77.3%)	0.455	181 (75.7%)	62 (72.9%)	119 (77.3%)	0.455
BP [n, (%)]	111 (46.6%)	41 (48.2%)	70 (45.8%)	0.713	111 (46.6%)	41 (48.2%)	70 (45.8%)	0.713
GLU [n, (%)]	107 (44.8%)	35 (41.2%)	72 (46.8%)	0.407	103 (43.1%)	35 (41.2%)	68 (44.2%)	0.656
Number of MS criteria								
0	8 (3.3)	3 (3.5)	5 (3.3)		8 (3.4)	3 (3.5)	5 (3.3)	
1	29 (12.1)	15 (17.6)	14 (9.2)		24 (10.1)	11 (12.9)	13 (8.5)	
2	72 (30.1)	21 (24.7)	51 (33.3)		60 (25.29	19 (22.4)	41 (26.8)	
3	67 (28.5)	23 (27.1)	44 (28.8)		70 (29.4)	22 (25.9)	48 (31.4)	
4	48 (20.1)	17 (20.0)	31 (20.3)		48 (20.2)	20 (23.5)	28 (18.3)	
5	14 (5.9)	6 (7.1)	8 (5.2)	0.411	28 (11.8)	10 (11.8)	18 (11.8)	0.718

Note - CL = Clinical lipodystrophy MS = Metabolic syndrome; ATPIII = Third Report of the National Cholesterol Education Program Expert Panel on Detection, Evaluation, and Treatment of High Blood Cholesterol in Adults; IDF = International Diabetes Federation; WC = waist circumference; TG = triglycerides; HDL = HDL cholesterol; BP = blood pressure; GLU = glucose.

**Table 6:** Frequency of each criterion of MS according to ATP III and IDF in women with and without clinical lipodystrophy

Women								
**ATPIII**					**IDF**			

**MS criteria**	**Total**	**Without CL**	**With CL**	**p**	**Total**	**Without CL**	**With CL**	**p**

WC [n, (%)]	61 (57.5%)	37 (68.5%)	24 (46.2%)	0.020	83 (78.3%)	45 (83.3%)	38 (73.1%)	0.200
TG [n, (%)]	78 (73.6%)	33 (61.1%)	45 (86.5%)	0.003	78 (73.6%)	33 (61.1%)	45 (86.5%)	0.003
HDL [n, (%)]	72 (67.9%)	31 (57.4%)	41 (78.8%)	0.018	72 (67.9%)	31(57.4%)	41 (78.8%)	0.018
BP [n, (%)]	37 (34.9%)	20 (37.0%)	17 (32.7%)	0.639	37 (34.9%)	20 (37.0%)	17 (32.7%)	0.639
GLU [n, (%)]	28 (26.4%)	16 (29.6%)	12 (23.1%)	0.444	28 (26.4%)	16 (29.6%)	12 (23.1%)	0.444
Number of MS criteria								
0	7 (6.6)	3 (5.6)	4 (7.7)	0.680	6 (5.7)	2 (3.7)	4 (7.7)	0.214
1	15 (14.2)	10 (18.5)	5 (9.6)		12 (11.3)	8 (14.8)	4 (7.7)	
2	34 (32.1)	16 (29.6)	18 (34.6)		26 (24.5)	16 (29.6)	10 (19.2)	
3	16 (15.1)	8 (14.8)	8 (15.4)		25 (23.6)	10 (18.5)	15 (28.8)	
4	25 (23.6)	14 (25.9)	11 (21.2)		26 (24.5)	15 (27.8)	11 (21.2)	
5	9 (8.5)	3 (5.6)	6 (11.5)		11 (10.4)	3 (5.6)	8 (15.4)	

Note - CL = Clinical lipodystrophy MS = Metabolic syndrome; ATPIII = Third Report of the National Cholesterol Education Program Expert Panel on Detection, Evaluation, and Treatment of High Blood Cholesterol in Adults; IDF = International Diabetes Federation; WC = waist circumference; TG = triglycerides; HDL = HDL cholesterol; BP = blood pressure; GLU = glucose.

Concerning the frequency of the individual ATPIII criteria for MS, we found a significantly lower prevalence of central obesity in men with CL and no other significant differences were observed. No significant differences were found in the frequency of the five IDF criteria for the MS in men (table 5).

In women with CL, using either ATPIII or IDF criteria, we found a statistically higher frequency of hypertriglyceridemia and lower HDL-C levels. According to ATPIII criteria, women with CL had a lower frequency of central obesity (Table 6).

No significant differences were found between the cumulative number MS features according to the presence of CL in both genders (Table 5 and 6).

In men, high blood pressure, high WC and the MS were more frequent in those with isolated central fat accumulation and mixed forms of lipodystrophy using either ATPIII or IDF criteria. High fasting glucose levels were significantly more frequent in patients with isolated central fat accumulation and mixed forms of lipodystrophy using ATPIII criteria and without any differences (p = 0.058) in the 4 groups of fat distribution using the IDF criteria (table 7).

**Table 7:** Metabolic syndrome and its individual components according the four different groups of fat distribution in men

	Four different groups of fat distribution				
**[n (%)]**	**No** **lipodystrophy**	**Isolated central** **fat accumulation**	**Isolated** **lipoatrophy**	**Mixed forms** **of lipodystrophy**	**P**

**ATP III**					
High blood pressure	17 (39.5)	24 (57.1)	36 (37.1)	33 (60.0)	0.017
Hypertriglyceridemia	32 (74.4)	33 (78.6)	79 (81.4)	52 (92.9)	0.086
Low HDL cholesterol	29 (67.4)	33 (78.6)	74 (76.3)	44 (78.6)	0.563
High waist circumference	0 (0.0)	21 (50.0)	0 (0.0)	22 (39.3)	< 0.001
High fasting glucose	13 (30.2)	22 (52.4)	40 (41.2)	32 (57.1)	0.035
Metabolic syndrome	16 (37.2)	30 (71.4)	43 (44.3)	40 (71.4)	< 0.001

**IDF**					
High blood pressure	17 (39.5)	24 (57.1)	36 (37.1)	33 (60.0)	0.017
Hypertriglyceridemia	32 (74.4)	33 (78.6)	79 (81.4)	52 (92.9)	0.086
Low HDL cholesterol	29 (67.4)	33 (78.6)	74 (76.3)	44 (78.6)	0.563
High waist circumference	0 (0.0)	42 (100.0)	0 (0.0)	56 (100.0)	< 0.001
High fasting glucose	13 (30.2)	22 (52.4)	38 (39.2)	30 (53.6)	0.058
Metabolic syndrome	0 (0.0)	36 (85.7)	0 (0.0)	52 (92.9)	< 0.001

In women, no significant differences were found in high blood pressure and high fasting glucose levels in the four groups of fat distribution (either ATPIII or IDF criteria). Hypertriglyceridemia, low C-HDL levels, high waist circumference and presence of the MS were more frequent in patients with isolated central fat accumulation and mixed forms of lipodystrophy using either ATPIII or IDF criteria (table 8).

**Table 8:** Metabolic syndrome and its individual components according the four different groups of fat distribution in women

	Four different groups of fat distribution				
**[n (%)]**	**No** **lipodystrophy**	**Isolated central** **fat accumulation**	**Isolated** **lipoatrophy**	**Mixed forms** **of lipodystrophy**	**P**

**ATP III**					

High blood pressure	1 (11.1)	19 (42.2)	2 (14.3)	15 (39.5)	0.110
Hypertriglyceridemia	4 (44.4)	29 (64.4)	8 (57.1)	37 (97.4)	< 0.001
Low HDL cholesterol	5 (55.6)	26 (57.8)	8 (57.1)	33 (86.8)	0.021
High waist circumference	0 (0.0)	37 (82.2)	0 (0.0)	24 (63.2)	< 0.001
High fasting glucose	2 (22.2)	14 (31.1)	1 (7.1)	11 (28.9)	0.335
Metabolic syndrome	0 (0.0)	25 (55.6)	1 (7.1)	24 (63.2)	< 0.001

**IDF**					

High blood pressure	1 (11.1)	19 (42.2)	2 (14.3)	15 (39.5)	0.110
Hypertriglyceridemia	4 (44.4)	29 (64.4)	8 (57.1)	37 (97.4)	< 0.001
Low HDL cholesterol	5 (55.6)	26 (57.8)	8 (57.1)	33 (86.8)	0.012
High waist circumference	0 (0.0)	45 (100.0)	0 (0.0)	38 (100.0)	< 0.001
High fasting glucose	2 (22.2)	14 (31.1)	1 (7.1)	11 (28.9)	0.348
Metabolic syndrome	0 (0.0)	28 (62.2)	0 (0.0)	33 (86.8)	< 0.001

Men and women with CL, after adjustment for age and cART, had significantly lower odds of having central obesity, as defined by the ATPIII criteria [OR = 0.403 (95%CI: 0.187-0.865) and OR = 0.306 (95%CI: 0.118-0.796), respectively].

In women, CL was significantly associated with higher odds of having MS as defined by IDF [OR = 5.290 (95%CI: 1.502-18.635)], hypertriglyceridemia [OR = 5.917 (95%CI: 1.50-23.349)] and low HDL cholesterol [OR = 3.990 (95%CI:1.252-12.716)], independently of age, cART and BMI (tables 9 and 10).

**Table 9:** Association between lipodystrophy and the metabolic syndrome and its individual components in men

	Model 1 OR (95% CI)	Model 2 OR (95% CI)	Model 3 OR (95% CI)
**MS ATP III**	1.017 (0.598-1.731)	0.621 (0.324-1.191)	0.931 (0.460-1.884)

High waist circumference	0.508 (0.260-0.991)	0.403 (0.187-0.865)	--
Hypertriglyceridemia	1.846 (0.940-3.624)	1.677 (0.782-3.600)	2.019 (0.899-4.531)
Low HDL cholesterol	1.261 (0.686-2.319)	1.116 (0.562-2.216)	1.304 (0.639-2.661)
High fasting glucose	1.254 (0.734-2.143)	1.004 (0.540-1.868)	1.256 (0.654-2.409)
High blood pressure	0.905 (0.532-1.540)	0.458 (0.223-0.939)	0.567 (0.270-1.191)
			

**MS IDF**	0.694 (0.402-1.196)	0.519 (0.274-0.985)	1.058 (0.473-2.365)

High waist circumference	0.585 (0.342-1.001)	0.491 (0.260-0.929)	--
Hypertriglyceridemia	1.846 (0.940-3.624)	1.677 (0.782-3.600)	2.019 (0.899-4.531)
Low HDL cholesterol	1.261 (0.686-2.319)	1.116 (0.562-2.216)	1.304 (0.639-2.661)
High fasting glucose	1.130 (0.661-1.931)	0.940 (0.506-1.743)	1.143 (0.599-2.180)
High blood pressure	0.905 (0.532-1.540)	0.458 (0.223-0.939)	0.567 (0.270-1.191)

Model 1 - OR crude

Model 2 - OR adjusted for gender, age and cART

Model 3 - OR adjusted for gender, age, duration of cART and BMI

**Table 10:** Association between lipodystrophy and the metabolic syndrome and its individual components in women

	Modelo 1 OR (95% CI)	Modelo 2 OR (95% CI)	Modelo3 OR (95% CI)
**MS ATP III**	1.074 (0.501-2.303)	0.763 (0.298-1.952)	2.730 (0.810-9.194)

High waist circumference	0.394 (0.178-0.869)	0.306 (0.118-0.796)	--
Hypertriglyceridemia	4.091 (1.557-10.750)	3.091 (1.063-8.989)	5.917 (1.50-23.349)
Low HDL cholesterol	2.765 (1.174-6.513)	2.308 (0.885-6.020)	3.990 (1.252-12.716)
High fasting glucose	0.713 (0.298-1.701)	0.560 (0.204-1.534)	0.758 (0.240-2.390)
High blood pressure	0.826 (0.371-1.839)	0.511 (0.177-1.474)	0.917 (0.273-3.078)
			

**MS IDF**	1.613 (0.742-3.507)	1.461 (0.572-3.736)	5.290 (1.502-18.635)

High waist circumference	0.543 (0.212-1.393)	0.385 (0.127-1.164)	--
Hypertriglyceridemia	4.091 (1.557-10.750)	3.091 (1.063-8.989)	5.917 (1.500-23.349)
Low HDL cholesterol	2.765 (1.174-6.513)	2.308 (0.885-6.020)	3.990 (1.252-12.716)
High fasting glucose	0.713 (0.298-1.701)	0.560 (0.204-1.534)	0.758 (0.240-2.390)
High blood pressure	0.826 (0.371-1.839)	0.511 (0.177-1.474)	0.917 (0.273-3.078)

Model 1 - OR crude

Model 2 - OR adjusted for gender, age and cART

Model 3 - OR adjusted for gender, age, duration of cART and BMI

No significant differences were found in the five classes of the Framingham risk score between patients with or without CL (data not shown).

Patients with CL had a significantly higher median risk of coronary heart disease at 10 years, measured by the Framingham risk score, than patients without CL, [median (25th and 75th percentile) = 7.00 (4.00-14.00) and 6.00 (4.00-11.00); p = 0.049]. Independent of the presence of CL, those with MS had a higher median of the Framingham risk score. In patients without CL, those with MS had a higher Framingham risk score median than those without MS, [median (25th and 75th percentile) = 7.00 (4.00-12.50) and 6.00 (3.00-9.00); p = 0.007]. Also, in patients with CL, those with MS, had a higher Framingham risk score than those without MS, [median (25th and 75th percentile) = 9.00 (6.00-18.00) and 6.00 (4.00-9.00); p < 0.001].

In patients with CL, we found significant differences in the Framingham risk score classes between patients with or without MS. Patients with CL and with MS had higher frequencies of moderate and high risk categories than those without MS (table 11).

**Table 11:** Framingham risk score according clinical lipodystrophy and metabolic syndrome

	Without clinical lipodystrophy			With clinical lipodystrophy		
**Framingham risk score**	**Without MS**	**With MS**	**Total**	**Without MS**	**With MS**	**Total**

Low risk (< 5%) [n (%)]	27 (45.8)	20 (29.4)	47 (37.0)	36 (41.9)	23 (21.5)	59 (30.6)
Average (5%-9%) [n (%)]	21 (35.6)	22 (32.4)	43 (33.9)	31 (36.0)	31 (29.0)	62 (32.1)
Moderate risk (10-19%) [n (%)]	7 (11.9)	13 (19.1)	20 (15.7)	15 (17.4)	31 (29.0)	46 (23.8)
High risk (20-39%) [n (%)]	3 (5.1)	12 (17.6)	15 (11.8)	2 (2.3)	20 (18.7)	22 (11.4)
Very high risk (> 40%) [n (%)]	1 (1.7)	1 (1.5)	2 (1.6)	2 (2.3)	2 (1.9)	4 (2.1)
p	0.078			< 0.001		

## Discussion

To our knowledge, this is the first study conducted in HIV-infected patients focusing specifically on the role of subcutaneous adipose tissue atrophy defined by presence of CL on the prevalence of the MS. Another particularity is that all patients were on cART. Other studies analysed the presence of the MS in naïve or on cART HIV-infected patients, with sub-analyses concerning the presence of CL in patients with the MS [12,14,17].

cART can induce a large spectrum of metabolic disturbances. On the other hand, lipodystrophy *per se*, including genetic lipodystrophy syndromes, is associated with lipid and metabolic disturbances, and hasmany similarities with the MS [2,31-35]. The effect of cART on the molecular processes of metabolic disturbances has been extensively studied [36] and its contribution to an increased risk of premature and accelerated atherosclerosis in HIV infection is well recognized [37,38]. Our aim was to determine the different patterns of metabolic alterations in patients with or without lipodystrophy.

It is unknown if the MS is more frequent in patients with lipodystrophy compared to either with patients without lipodystrophy and not HIV-infected [9,17]. We found a higher prevalence of MS compared with some studies [9,10,12-23] but similar to another [11]. We included all patients referred from the Infectious Diseases Department to our Endocrinology Out-patient Clinic for metabolic abnormalities, but we cannot exclude the selection bias by the infectious disease specialist. We found a high prevalence of lipodystrophy but there was no selection bias for the presence of isolated lipoatrohy or abdominal prominence. Since our main goal was to evaluate whether the presence of lipodystrophy influences MA, only patients on cART were included. The high MS prevalence may reflect the fact that all the patients were on cART. Another important fact is the low prevalence of intravenous drug users in our study (24.6% in patients without CL and 30.6% in patients with CL) compared with Jericó study (41.3%), which concurred on the low prevalence of abdominal obesity.

Studies of prevalence of MS in HIV-infected individuals compared with control populations revealed different prevalences of MS) [9-23] and it also seems to be higher than the 22 to 24% prevalence rate reported for the general US population [39]. Other data suggest that the increased prevalence of MS among HIV-infected persons may be reflective of the burgeoning epidemic of obesity than a predominant effect of cART [40]. Cigarette smoking analysis could be important because of its frequent association with lower BMI and higher glucose levels, however, no significant difference was found in smoking history (never, current or former) between patients with or without CL. Also, no significant difference was found in fasting plasma glucose levels according the smoking history.

In our sample, the prevalence of MS was lower using the IDF definition compared to the ATPIII criteria either in the total of the patients or in men with or without CL. On the other hand, in women it was higher when the IDF definition was used. This probably mirrors the fact that central obesity is a compulsory criterion for the IDF definition, and, women had a higher mean of WC. Since HIV patients despite lower WC had increased visceral fat, Capeau J proposed that a different WC cut-off is needed for HIV patients [34]. In fact, in our data, patients with CL after adjustment for age and cART had significantly lower odds of having central obesity.

No differences in the prevalence of ATPIII or IDF defined MS between patients with and without CL were observed, like it was previously described by Estrada [14]. In women, CL was significantly associated with MS, hypertriglyceridemia and low HDL cholesterol independently of age, cART and BMI. Analyzing the data differently, Samaras showed that the prevalence of CL was 57.2% in the total of patients, and 73% and 79% in those with MS defined by respectively IDF and ATPIII criteria [17]. In our patients, we did not find any significant differences in the prevalence of lipodystrophy in patients with and without MS, according to both criteria. In the Jericó study, as in Samaras, lipodystrophy was more common among MS participants (50.4 vs 33.8; p = 0.0001) [12], and Squillace, also found that CL was strongly associated with MS [22].

In the Jericó and Mangili studies, the prevalence of MS in HIV-infected patients significantly increased with age [12]. In our sample, we did not observe any differences in the distribution of MS prevalence according to age. This may reflect the homogeneity of age strata in our sample, when compared to other studies (only 12% of our sample were 60 years or older).

Regarding gender differences, as in our study, Mangili reported that significantly more women than men had MS [10].

Concerning the number of MS components, in our study and according to IDF definition, we had 25% of patients with two features of MS which is lower when compared to other studies [12,17]. In the Samara study, 49% of patients had at least two features of MS, namely elevated lipid levels [17]. In the Jericó study, the MS prevalence was 17%, with 69.3% of patients showing one or more features of MS, 35.8% two or more, 4.5% three or more and 0.1% five features [12].

Concerning the prevalence of the individual features of MS, we found that the two least prevalent features were high fasting glucose level (similar to Bonfatti and Sobieszczyk) [18,21] and high WC, the latter being the least prevalent in the patients with CL. Lipid abnormalities, namely high triglyceride and low HDL-C levels, were present in all HIV-infected patients [9,10,12,13,16,17] and also in CL patients, the most prevalent components of MS. Obesity is an important risk factor for MS in the general population and in HIV-infected individuals [40]. We found a higher prevalence of overweight and obesity among the patients without CL. Although not proving causation, because of the cross-sectional nature of the study design, we can hypothesize that the high frequency of overweight and obesity in patients without CL may contribute to the observed absence of difference in the MS prevalence between patients with or without CL. To clarify the relation between obesity and MS as well as the alterations in the fat distribution, data was analysed according to the presence or absence of either lipoatrophy or abdominal prominence. In men, high blood pressure and MS were more frequent in those with isolated central fat accumulation and mixed forms of lipodystrophy (ATPIII and IDF criteria) and high fasting glucose levels were significantly more frequent in same two groups using ATPIII criteria. In women, hypertriglyceridemia, low C-HDL levels, high WC and presence of MS were more frequent in those with isolated central fat accumulation and mixed forms of lipodystrophy using either ATPIII or IDF criteria. The observation that patients with CL had significantly higher risk of coronary heart disease at 10 years, measured by the Framingham risk score, than patients without CL and that those with CL and with MS had higher frequencies of moderate and high risk categories than those without MS emphasizes the importance of the clinical diagnosis of this condition. The higher risk of CHD at 10 years of HIV lipodystrophic patients was previously reported by Ena [41]. In the De Socio study, a correlation between the Framingham risk score and MS was observed, and patients with lipodystrophy had a higher Framingham risk score than patients without lipodystrophy, suggesting that the presence of lipodystrophy has metabolic implications equivalent to those of MS [42].

Our study had some limitations. We did not exclude patients with diabetes or hypertension diagnosed prior to HIV infection. We also cannot discard the influence of the pre-HIV body composition, the cumulative exposure time of each drug and the nadir value of CD4 factors that could contribute to the risk lipoatrophy or abdominal prominence that were not evaluated. It is difficult to compare results on the prevalence of MS because of the heterogeneity in populations regarding past and actual cART regimens and treatment adherence. Regarding the CD4 count we only presented the values at the time of the cross sectional evaluation, because we did not have access to the nadir value. Finally, there exists the possibility of selection bias. Some subjects in this study were referred from to Endocrinology Clinics specifically for lipodystrophy or metabolic disorders related to cART, and so we might have selected for a study population enriched for metabolic complications.

## Conclusions

In women, CL was significantly associated with the metabolic syndrome, hypertriglyceridemia and low HDL cholesterol independent of age, cART and BMI.

Our data showed that among HIV patients on cART, the prevalence of MS may be remarkably high, especially when taking into account the mean age of our sample. The observation that patients with CL had a significantly higher risk of coronary heart disease at 10 years, measured by the Framingham risk score, than patients without CL and those with CL and with MS had higher frequencies of moderate and high risk categories than those with CL but without MS, emphasizes the importance of the clinical diagnosis of this condition.

## List of abbreviations

AHA/NHLBI: American Heart Association/National Heart, Lung, and Blood Institute Scientific Statement; ATPIII: The Third Report of Expert Panel on Detection, Evaluation, and Treatment of High Blood Cholesterol in Adults; BP: Blood pressure; cART - Combination antiretroviral therapy; CL: Clinical lipodystrophy; CVD: Cardiovascular disease; HIV: Human immunodeficiency virus; IDF: International Diabetes Federation; MA: Metabolic abnormalities; MS: Metabolic syndrome; NCEP: National Cholesterol Education Program; WC: Waist circumference.

## Competing interests

The authors declare that they have no competing interests.

## Authors' contributions

PF conceived the study, participated in its design, in the acquisition of data and drafted the manuscript; DC conceived the study, participated in its design and drafted the manuscript; SS participate in the acquisition of data and drafted the manuscript; SX participate in the acquisition of data; ACS performed the statistical analysis and revised critically the manuscript; RM participated in its design and revised critically the manuscript; EM and AS revised critically the manuscript; JLM revised the study design. All authors read and approved the final manuscript.

## Funding

Research Fellowship Dr. Manuel Almeida Ruas, Portuguese Society of Diabetology. Research Fellowship of the Portuguese Association for Clinical Study of AIDS. Research Grant to support doctoral studies in the area of HIV/AIDS Foundation GlaxoSmithKline of Health Sciences.

## Pre-publication history

The pre-publication history for this paper can be accessed here:

http://www.biomedcentral.com/1471-2334/11/246/prepub
